# Omental Torsion Diagnosed and Treated with Single-Incision Laparoscopic Surgery in 2 Pediatric Patients: A Case Report

**DOI:** 10.70352/scrj.cr.24-0021

**Published:** 2025-01-31

**Authors:** Shohei Maekawa, Masafumi Kamiyama, Chisato Fujita, Daishi Takao, Kiyoaki Sumi, Kimihiko Watanabe, Kazunori Masahata

**Affiliations:** 1 Department of Pediatric Surgery, Aizenbashi Hospital, Osaka, Osaka, Japan; 2 Department of Pediatric Surgery, Osaka University Graduate School of Medicine, Suita, Osaka, Japan; 3 Department of Pediatrics, Aizenbashi Hospital, Osaka, Osaka, Japan; 4 Department of Internal Medicine, Aizenbashi Hospital, Osaka, Osaka, Japan

**Keywords:** omental torsion, acute abdominal pain, pediatrics

## Abstract

**INTRODUCTION:**

Omental torsion (OT), caused by twisting of the greater omentum around its axis, leading to reduced blood supply to the distal aspect of the omentum and tissue infarction, is a rare disease that manifests clinically as acute abdominal pain. Accurate preoperative diagnosis is difficult. Here, we present 2 pediatric patients diagnosed and treated using computed tomography (CT).

**CASE PRESENTATION:**

Case 1, a 14-year-old boy, had abdominal pain for 3 days. Upon referral to our hospital due to worsening pain, CT revealed an intra-abdominal fatty mass extending into high-density lesions in the fat tissue. Due to severe peritoneal irritation, emergency single-incision laparoscopic surgery was performed. Secondary OT was diagnosed as the greater omentum was twisted by the cord-like tissue, continuing from the greater omental infarction to the lesser omentum. Partial omentectomy, including the ischemic portion, was performed. Case 2, an 11-year-old boy, was referred with suspected appendicitis due to right lower abdominal pain for 2 days. CT revealed a whirling sign in the greater omentum and high-density lesions in the fat tissue. The patient was in good condition, and the peritoneal irritation was unclear; therefore, conservative treatment was initiated. However, symptoms did not improve after 48 h and single-incision laparoscopic surgery was performed, revealing a twisted necrotic omental mass diagnosed as primary idiopathic greater OT. Partial omentectomy, including the ischemic portion, was performed.

**CONCLUSIONS:**

CT scan aids in preoperative diagnosis of OT, for which single-incision laparoscopic surgery is a less invasive and useful therapy. Early surgical intervention is warranted when conservative treatment fails.

## Abbreviations

OT
: omental torsion
CT
: computed tomography
WBC
: white blood cell
CRP
: C-reactive protein

## INTRODUCTION

Omental torsion (OT) is a rare cause of acute abdominal pain in children, which develops when the omentum twists and its periphery becomes ischemic.^1)^ Since the first report of OT by Eitel et al. in 1899,^2)^ approximately 400 cases have been reported, of which only 15% were reported in children.^1)^ OT has no characteristic clinical findings, with clinical symptoms resembling those of acute appendicitis, making it difficult to diagnose it before treatment^3,4)^ correctly. Although OT is accurately diagnosed preoperatively in only <5% of cases, it can be accurately diagnosed using computed tomography (CT).^5)^

Herein, we present 2 pediatric patients preoperatively diagnosed with OT based on CT findings and successfully treated with single-incision laparoscopic surgery.

## CASE PRESENTATION

### Case 1

**Fig. 1:**
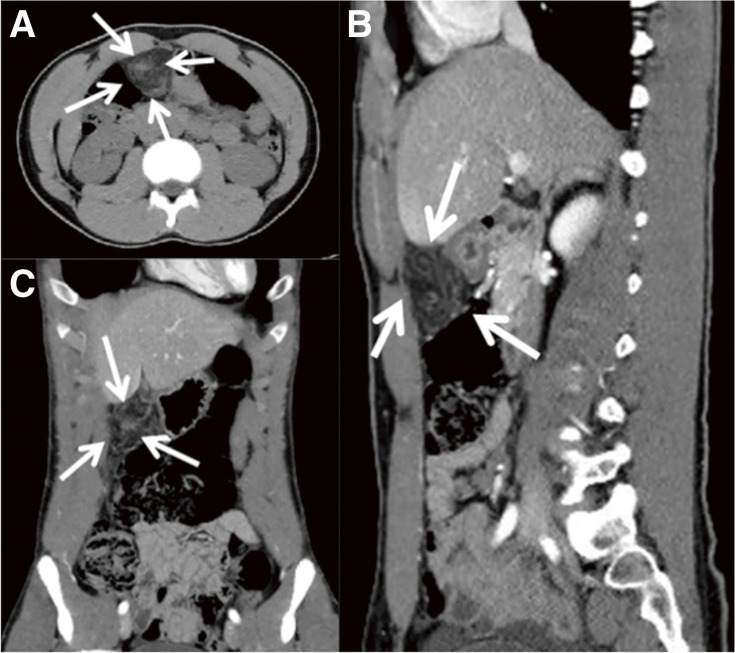
CT results of Case 1. (**A**) Transverse plane. (**B**) Sagittal plane. (**C**) Coronal plane. Whirl sign of omental torsion and increased fat concentration on CT (white arrow). CT, computed tomography

A 14-year-old boy was referred to our hospital with abdominal pain, which was suspected to be worsened gastritis. The patient had experienced abdominal pain for 3 days prior to admission, which was so severe that there was no relief even after taking an H2-blocker. The patient had severe tenderness and rebound pain, predominantly on the right side of his abdomen, and was unable to lie down, requiring constant bending of his body. He had no other relevant symptoms or history of surgery. Assessment of vital signs at admission revealed a pulse rate of 69 beats/min, normal blood pressure of 122/62 mmHg, and a slight fever (37.7°C). Physical examination revealed a height of 162.0 cm, a weight of 55.0 kg, and a body mass index (BMI) of 21.0 kg/m^2^. Blood tests revealed a white blood cell (WBC) count of 6900 μL with 72.7% neutrophils, 18.9% lymphocytes, 6.2% monocytes, 1.6% eosinophils, and 0.6% basophils, and an elevated C-reactive protein (CRP) level of 3.30 mg/dL. Abdominal CT revealed axle-shaped omental vessels, a spiral omentum, and an increased concentration of fat in the twisted omentum (**Fig. 1**). Inguinal hernia and appendiceal swelling were not observed. OT was diagnosed, and emergency transumbilical single-incision laparoscopic surgery was performed.

**Fig. 2:**
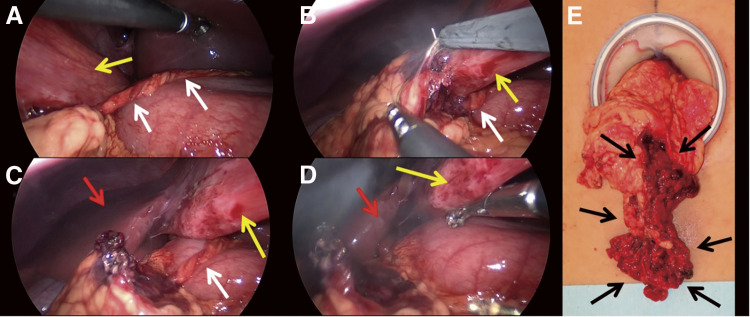
Intraoperative findings of Case 1. (**A**) A cord-like tissue (white arrow) extending from the twisted greater omentum to the lesser omentum was observed. (**B**) The adhesion of the twisted omentum to the falciform ligament (yellow arrow) had become detached. (**C**) The adhesion of the twisted omentum to the inferior liver (red arrow) had become detached. (**D**) The twisted omentum was mobilized by cutting the cord-like tissue. (**E**) The twisted omentum (black arrows) was removed from the abdominal cavity via an umbilical incision.

The twisted greater omentum was laparoscopically observed inferior to the liver on the right upper side of the abdominal cavity. A cord-like tissue was observed to extend from the twisted greater omentum to the lesser omentum, with the twisted greater omentum acting as an axis. Funicular tissue formed part of the greater omentum that adhered to the lesser omentum. Partial omentectomy, including the torsional omentum, was performed (**Fig. 2**). The histological examination revealed congestive changes, evidence of tissue infarction due to torsion, and no apparent neoplastic lesions.

The patient was discharged from the hospital 4 days postoperatively without any complications. No signs of recurrence were observed 3 months after the operation.

### Case 2

**Fig. 3:**
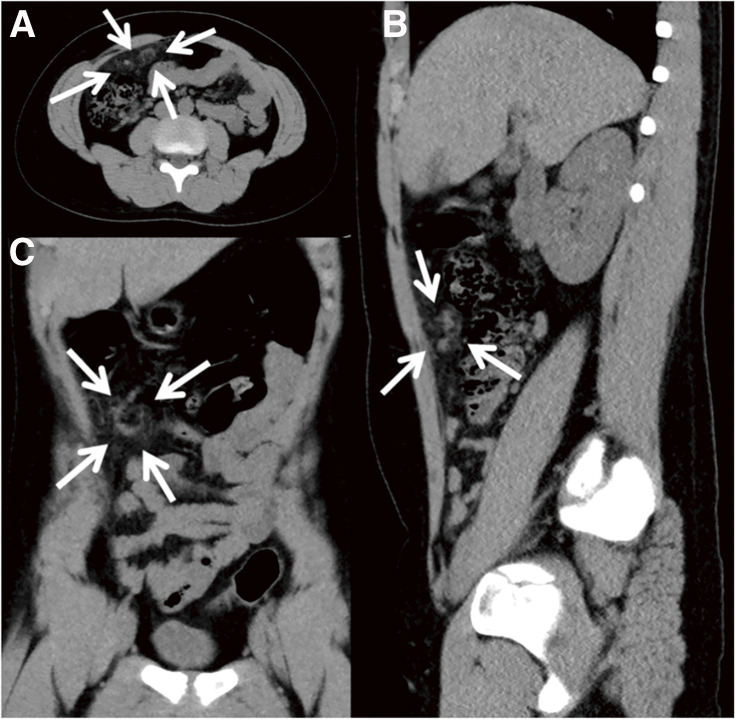
CT results of Case 2. (**A**) Transverse plane. (**B**) Sagittal plane. (**C**) Coronal plane. Whirl sign of omental torsion and increased fat concentration on CT (white arrows). CT, computed tomography

An 11-year-old boy with a 2-day history of abdominal pain suspected of having acute appendicitis was referred to our hospital. The patient had not experienced any similar episodes of abdominal pain. Physical examination revealed a height of 135.3 cm, weight of 40.2 kg, and BMI of 22.0 kg/m^2^. Abdominal pain and tenderness near the navel were localized to the right side of the hemiabdomen, cranial to McBurney’s point. The patient had no associated nausea or vomiting. He appeared to be in pain and had mild tachycardia with a pulse rate of 91 beats/min, normal blood pressure of 104/71 mmHg, and no fever (36.7°C). Blood tests revealed a WBC count of 9100 μL with 75.9% neutrophils, 12.9% lymphocytes, 6.5% monocytes, 4.1% eosinophils, and 0.6% basophils. Other than a slightly increased CRP level of 0.58 mg/dL, there were no notable findings. A CT scan of the abdomen revealed a mass-like lesion on the ventral side of the ascending colon, with localized increased density of the surrounding fat tissue in the greater omentum and spiral-shaped omental blood vessels (**Fig. 3**). There were no findings of diverticulitis in the ascending colon or appendicitis, and only a small amount of ascites was observed in the pelvic cavity.

The patient was subsequently admitted to our hospital and diagnosed with OT. Initially, his vital signs were stable, and his symptoms were nonspecific and mild; therefore, he was treated conservatively; however, after 48 h, his abdominal pain worsened, and localized peritoneal irritation symptoms appeared. A decision was subsequently made to perform transumbilical single-incision laparoscopic surgery.

**Fig. 4:**
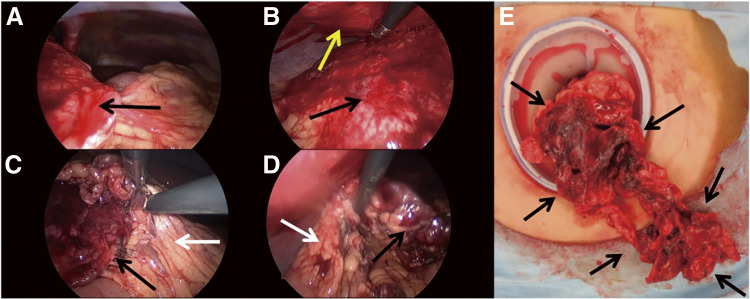
Intraoperative findings of Case 2. (**A, B**) The adhesion of the twisted omentum (black arrow) to the abdominal wall (yellow arrow) had become detached. (**C**) The adhesion of the twisted omentum to the normal omentum (white arrow) had become detached. (**D**) The twisted omentum was completely mobilized. (**E**) The twisted omentum was removed from the abdominal cavity via an umbilical incision.

Laparoscopy revealed a twisted omentum on the right side of the abdominal cavity, with inflammatory adhesions to the abdominal wall. When these inflammatory adhesions were removed, local peritonitis was observed. The twisted omentum was removed from the abdominal cavity through an umbilical incision, while a partial omentectomy was performed (**Fig. 4**). The patient was discharged 3 days later without complications. Histological examination revealed severe congestion and purulent changes, with neutrophil infiltration into the adipose tissue. There was no evidence of massive lesions, confirming the diagnosis of OT. No signs of recurrence were observed 3 months following the operation.

## DISCUSSION

OT is a rare condition in which part of or the entire greater omentum is under torsion, resulting in impaired blood flow to the peripheral side, abdominal pain, and other symptoms. Although adult cases have been occasionally reported, particularly in middle-aged men, pediatric cases are rare, accounting for only approximately 15% of all cases.^1)^ It has been speculated that because the omentum is relatively underdeveloped in children compared to adults, the risk of torsion is anatomically lower.^6)^ The reported male:female ratio of OT is 2:1.^7)^ OT is classified as either primary or secondary,^8)^ depending on the presence or absence of organic lesions, such as causative adhesions, hernias, or tumors.^9,10)^ The cause of OT is unclear; however, it is thought to occur due to a confluence of factors such as anatomical abnormalities of the greater omentum, positional relationship of the arteries and veins within the greater omentum, obesity, increased intestinal peristalsis, and sudden changes in position. However, neither of the 2 patients in our study were obese. In Case 1, adhesions of the greater omentum to the lesser omentum were found during the operation, and the patient was diagnosed with secondary OT due to adhesions. By contrast, Case 2 had no history of trauma or overeating, and no cause of increased intestinal peristalsis; therefore, the patient was diagnosed with primary OT.

The difficulty in diagnosing OT is thought to be due to the infrequency of the disease and lack of specificity in the symptoms associated with OT. The incidence rate is reported to be 0.0016%–0.37%, accounting for 1.1% of all cases of acute abdominal pain.^5)^ Because most patients present with right lower abdominal pain, it is challenging to distinguish OT from acute appendicitis.^3,4,11)^ According to prior reports, the estimated incidence of OT in children undergoing laparotomy for suspected appendicitis varies between 0.024% and 0.1%.^12)^ The right-side omentum is longer than the left-side omentum, making it more mobile; consequently, it is more common for the right side to be twisted along its blood supply, causing OT.^13)^ Consequently, left-side OT is extremely rare.^14)^ The symptoms of OT differ from acute appendicitis in that it is rarely accompanied by gastrointestinal symptoms, such as nausea and vomiting, while symptoms of severe peritoneal irritation, fever, elevations in the WBC count, and inflammatory responses are mild.^1,3,4,11)^ In our case, both patients had abdominal pain but no gastrointestinal symptoms. The CRP levels of Cases 1 and 2 were 1.40 and 0.58 mg/dL, respectively, at the time of their first visit to our hospital; however, the WBC levels in both cases were within the normal range. Therefore, the symptoms and blood test results alone were insufficient to diagnose OT.

Following the first report of preoperative diagnosis of OT using CT by Ceuterick et al. in 1987,^15)^ CT is now considered useful in facilitating an accurate preoperative diagnosis of OT.^1,16–18)^ Due to its anatomical characteristics, the omentum in the torsional area is depicted on CT as a “fat density mass” with a low-density area compared to the parenchymal organs and intestines and a slightly higher density area than the mesentery. Within the fat density mass, a “whirl sign” shows a spiral or concentric layered structure with a mixture of high- and low-density areas.^16–18)^ Ceuterick et al. described these characteristics as “a large fat density mass with linear strands in concentric pattern.”^15)^ As cases of OT without the “whirl sign” have also been reported,^19)^ it is necessary to observe the continuous CT images to evaluate whether or not torsion has occurred. Despite the characteristic CT findings, preoperative diagnosis is difficult, and it has been reported that only approximately 10% of cases are diagnosed preoperatively. In 1 prior study, Chen et al.^1)^ reported that of 12/17 (70.6%) pediatric cases of OT were diagnosed preoperatively using CT, highlighting its usefulness. When a patient presents with right lumbar or right lower abdominal pain, OT must also be considered in the differential diagnosis if acute appendicitis is not actively suspected based on the medical history and clinical symptoms. CT should be performed immediately if OT is suspected in the differential diagnosis. In both of the present cases, CT was performed, allowing a correct diagnosis of OT and demonstrating the importance of CT in OT diagnosis.

The treatment of OT involves either surgery or conservative treatment.^13,20)^ Although there have been reported cases in which the condition improved without surgery, there are also cases, such as Case 2, in which surgery was required. In Case 2, conservative treatment was initially selected because the patient was in good general condition with no peritoneal irritation symptoms at the time of diagnosis. However, because his symptoms did not improve after 48 h of observation, surgery was ultimately performed. As both patients were discharged around 3 days after surgery, we considered that conservative observation, which would prolong the disease, should not be continued. This operation should be performed if symptoms do not improve after 24–48 h of observation.^13,20)^ Laparoscopic surgery is considered to be the best surgical option. First, laparoscopic examination of the abdominal cavity enables a detailed diagnosis of the cause of secondary OT, while treatment of the causative disease can be performed with minimal invasiveness. In particular, single-incision laparoscopic surgery via an intraumbilical incision has the advantage of allowing for a hybrid of laparoscopic and extracorporeal procedures, even in pediatric cases in which the intraperitoneal space is small, as the greater omentum can be easily guided out of the body. In both patients, the disease was diagnosed using diagnostic laparoscopy. In Case 1, the adhesion tissue causing torsion was treated inside the abdominal cavity. The omentum was removed from the abdominal cavity, while the torsional area was resected along with the normal tissue. These procedures were performed without difficulty. As such, it is considered a useful method as it allows for a safe operation, short postoperative hospitalization, and high wound satisfaction.

## CONCLUSION

Although OT is an extremely rare disease, it should be considered a differential diagnosis in pediatric cases of acute abdominal pain. Abdominal CT is beneficial for the diagnosis of OT; when a definitive diagnosis is made, surgery or conservative treatment should be considered, depending on the patient’s general condition. Surgery should also be considered within 48 h of no response to conservative treatment. Single-incision laparoscopic surgery is minimally invasive and enables the accurate diagnosis and treatment of the OT of children.

## ACKNOWLEDGMENTS

We would like to thank Editage (www.editage.com) for editing the English language.

## DECLARATIONS

### Funding

All authors declare that they have no conflicts of interest.

### Authors’ contributions

SM drafted the manuscript.

SM, MK, CF, DT, and KS diagnosed and treated in Case 1.

SM, KS, KW, and KM diagnosed and treated in Case 2.

SM and MK performed the surgery for Case 1.

SM and KM performed the surgery for Case 2.

KM critically revised the manuscript.

All authors have read and approved the final version of the manuscript.

### Availability of data and materials

The datasets analyzed in the current study are available from the corresponding author upon reasonable request.

### Ethical approval and consent to participate

This case report was conducted according to the principles of the Declaration of Helsinki, and the confidentiality of records identifying the subject to be maintained was given oral explanations, and consent was obtained in advance.

### Consent for publication

Informed consent was obtained from the patients for publication of this case report.

### Competing interests

The authors declare that they have no competing interests.
